# Aetiology of community-acquired, acute gastroenteritis in hospitalised adults: a prospective cohort study

**DOI:** 10.1186/1471-2334-8-143

**Published:** 2008-10-22

**Authors:** Andreas Jansen, Klaus Stark, Jan Kunkel, Eckart Schreier, Ralf Ignatius, Oliver Liesenfeld, Dirk Werber, Ulf B Göbel, Martin Zeitz, Thomas Schneider

**Affiliations:** 1 Department for Infectious Disease Epidemiology, Robert Koch Institute, Berlin, Germany; 2 Medical Clinic I, Campus Benjamin Franklin, Charité, Berlin, Germany; 3 Department for Molecular Epidemiology of Viral Pathogens, Robert Koch Institute, Berlin, Germany; 4 Department of Microbiology and Hygiene, Charité, Berlin, Germany

## Abstract

**Background:**

The aetiology of severe gastroenteritis leading to hospitalisation in adults frequently remains unclear. Our objective was to study the causes and characteristics of community-acquired, acute gastroenteritis in adult hospitalized patients to support the clinical management of these patients.

**Methods:**

From August 2005 to August 2007, we conducted a prospective cohort study among patients ≥18 y hospitalized with community-acquired gastroenteritis in a university hospital in Berlin, Germany. Stool specimens were examined for 26 gastrointestinal pathogens, supplemented by serologic tests for antibodies to *Campylobacter spp.*, *Yersinia spp.*, and *Entamoeba histolytica*. Patient data on demographics and clinical presentation were recorded and analyzed. Coexisting medical conditions were assessed using the Charlson Comorbidity Index score.

**Results:**

Of 132 patients presenting with acute community-acquired gastroenteritis, 104 were included in the study. A non-infectious aetiology was diagnosed in 8 patients (8%). In 79 (82%) of the remaining 96 patients at least one microorganism was identified. *Campylobacter spp. *(35%) was detected most frequently, followed by norovirus (23%), *Salmonella spp. *(20%), and rotavirus (15%). In 46% of the patients with *Campylobacter spp. *infection, the diagnosis was made solely by serology. More than one pathogen was found in seventeen (22%) patients. Simultaneous infection was significantly more likely in patients with rotavirus and salmonella infections (RR 3.6; 95% CI: 1.8–7.4; RR 2.5; 95%CI: 1.2–5.5). Length of hospital stay (median: 5.5 days) was independent of the pathogen, but was associated with coexisting medical conditions (OR 4,8; 95%CI:2,0–11,6).

**Conclusion:**

Known enteric pathogens were detected in 82% of adult patients who were hospitalized with acute gastroenteritis. We found that currently used culture-based methods may miss a substantial proportion of *Campylobacter *infections, and additional serological testing for *Campylobacter *should be considered. Viral infections emerged as an important cause of severe gastroenteritis in adults, and viral-bacterial co-infections in adults are probably underrecognized so far. The presence of coexisting medical conditions – but not the etiological agent – was a predictor for the duration of the hospital stay.

## Background

Infectious gastroenteritis is a leading cause of morbidity and mortality worldwide [1]. In developed countries, peak incidences of infectious gastroenteritis are found in younger age groups (< 5 years), while severe disease leading to hospitalisation and resulting in death is most frequently observed in elderly patients (> 60 years) [2]. Several studies have focused on the aetiology of infectious diarrhea in hospitalized children. The epidemiology of hospitalisations associated with gastroenteritis in adults, however, has not been well investigated so far, even though it imposes a major burden on the patient and the health care system [3]. In a few, mostly retrospective studies a causative pathogen was diagnosed in only 39–58% of all patients, leaving a considerable diagnostic gap [4-7].

The objective of the present study was to investigate the aetiology and characteristics of community-acquired, acute gastroenteritis leading to hospitalisation in adults in a developed country. Our intention was also to provide data for recommendations concerning routine testing panels in this patient group to guide clinicians in the rational use of diagnostic methods for enteric pathogens. In addition, since hospitalisation for gastroenteritis is generally considered to be a preventable outcome, we determined possible risk groups for severe disease to put forward targeted preventive measures.

## Methods

### Study design

From August 2005 to August 2007, we conducted a prospective cohort study among patients ≥18 years of age presenting with acute gastroenteritis as the primary diagnosis at the emergency department of the Charité – Benjamin Franklin University Hospital and who were subsequently referred to the infectious disease ward. Reasons for hospitalisation included severe dehydration, and other conditions requiring stationary treatment (e.g. severe hypocalemia, new-onset atrial fibrillation, anaemia). The hospital with 1030 hospital beds, and 19 medical departments is located in the south-west of Berlin, and serves a demographically varied urban and suburban population. Patients were considered to have acute gastroenteritis if they had ≥ 3 loose stools per day or vomited, and their onset of symptoms was within 48 h before presentation. Patients were excluded from the study if a history of inflammatory bowel disease (Crohn's disease or ulcerative colitis) or of other diseases associated with diarrhea (e.g., celiac disease, collagenous or microscopic colitis, misuse of laxatives, Whipple's disease, irritable bowel syndrome, or chronic pancreatitis) was known. Information on demographics (age, sex, place of residence), clinical presentation (onset of symptoms, stool frequency), and the medical history for each patient was captured on a standard structured questionnaire. Coexisting medical conditions were assessed using the Charlson Comorbidity Index score, including 19 major disease categories [8,9].

The study was approved by the ethical committee of the Charité, and all study participants had given their written, informed consent.

### Stool specimens and serum samples

At least three separate stool specimens for bacterial culture and one stool specimen for viral reverse transcriptase polymerase chain reaction (RT-PCR) were provided by all patients and subjected to microbiological analysis. The first stool specimens for each patient were collected within 24 hours after admission.

Patients for whom stool cultures did not yield positive results were asked to provide paired serum samples to determine antibodies to *Campylobacter spp.*, *Yersinia spp.*, and *Entamoeba (E.) histolytica*. The first serum sample was collected within the first five days and a second one between the third and sixth week after the onset of the disease.

### Detection of bacteria

For the growth of *Clostridium (C.) difficile*, stool specimens were pre-treated with alcohol shock (absolute alcohol for 30 min) before culture. Otherwise, fresh stool specimens were used. Standard solid media, i.e., MacConkey, xylose lysine deoxycholate, bismuth sulfite (Wilson-Blair), and Salmonella-Shigella agars, as well as selenite and tetrathionate (Preuss) broths (all Oxoid, Wesel, Germany) were used to detect *Salmonella, Shigella, Aeromonas, Plesiomonas, and Vibrio species*. In addition, cefsulodin irgasan novobiocin agar, Skirrow agar supplemented with 10% sheep blood, PALCAM listeria agar, and cycloserine-cefoxitin fructose agar (all Oxoid) were inoculated for the detection of *Yersinia spp., Campylobacter spp., Listeria monocytogenes*, and *C. difficile*, respectively. To detect *Arcobacter butzleri*, Skirrow agar plates were inoculated and cultured at 37°C. Suspicious bacterial colonies were further isolated and differentiated using routine techniques (e.g., oxidase, hippurate, and motility for *Campylobacter spp. *and *A. butzleri*, agglutination for *Salmonella spp.*, *Shigella spp.*, and *Vibrio spp.*) and the API system (BioMerieux, Nürtingen, Germany). For the detection enterohemorrhagic *Escherichia coli *(EHEC) pre-enriched cultures of stool specimens were tested with a Shiga toxin EIA.

To detect mycobacteria, stool specimens were decontaminated with NaOH and N-acetyl-L-cysteine and cultured on Stonebrink and Loewenstein-Jensen agar (BAG, Lich, Germany) and in a liquid broth culture system (Becton Dickinson, Heidelberg, Germany). Positive samples were then confirmed by use of PCR techniques. Growth of mycobacteria was confirmed by acid-fast staining and the isolates were differentiated by commercially available nucleic acid probe- and amplification-based systems.

*C. difficile *toxin was detected in stool samples using a commercially available ELISA assay (Microtest, Mainz, Germany).

### Serology

Antibodies to *Campylobacter spp. *were detected by performing complement fixation tests (CFT) (Virion/Serion, Würzburg, Germany), and positive results were defined by a ≥ 3-fold increase in antibody titres between paired serum samples. Antibodies to *Yersinia spp. *were detected by agglutination tests (Sanofi Diagnostics Pasteur, Marnes-La-Coquette, France). The specificity was confirmed by western blot (Genzyme Virotech, Rüsselsheim, Germany). To detect antibodies to *E. histolytica *antigens, indirect fluorescence antibody (BioMerieux, Nürtingen, Germany) and indirect haemagglutination tests (Dade Behring, Marburg, Germany) were performed.

### Detection of viruses

Six viruses (i.e., norovirus, rotavirus, aichivirus, adenovirus, astrovirus, and enteroviruses) were detected by RT-PCR techniques as previously described [10].

### Microscopic detection of parasites

Fresh stool specimens were enriched by the SAF fixation-concentration technique. Wet mounts were prepared from the sediments and analyzed for trophozoites, cysts, or oocysts, respectively, of *Giardia lamblia*, *Cyclospora cayetanensis, Isospora belli, E. histolytica/dispar, Balantidium coli*, and *Blastocystis hominis*, as well as for helminth eggs. Additionally, a direct immune fluorescence antibody test (Genzyme Virotech) was performed to detect cysts of *G. lamblia *and oocysts of cryptosporidia. Moreover, air-dried slides were stained according to standard protocols by using the Kinyoun method (BioMerieux, France) for the detection of acid-fast coccidia (cryptosporidia, *C. cayetanensis, I. belli*) and following the Uvitex method for the detection of microsporidia, respectively.

### Statistical analysis

For single proportions, 95% confidence intervals (95% CI) were calculated according to Wilson. One-way ANOVA, Student's t-test, and the Mann-Whitney test were used for comparative analysis of continuous variables. The Pearson's χ^2 ^test or Fisher's exact test were used to assess the significance of differences in proportions between groups. For cross-tabulations, continuous variables (e.g., length of hospital stay) were dichotomized using the mean as cut-off. Logistic regression analysis was used to identify risk factors for length of hospital stay. Variables were manually offered to the model based on their p-value in log-likelihood tests. Final models were checked for interaction terms and colinearity. Relative risks (RR), Mantel-Haenzel odds ratios (OR_MH_), and 95% confidence intervals (CI) were calculated using SPSS 14 software, Chicago, USA. A p-value of < 0.05 was considered significant.

## Results

### Study cohort

Of all 132 patients hospitalized with acute diarrhea during the study period, 104 (79%) gave their consent and were included in the study. Twenty-eight patients refused to participate or were mentally unable to sign the consent form. The median age of study patients was 48 (18–91) years, and 47 (45%) were males. Patients were admitted throughout the year with peak admissions for patients infected by bacterial pathogens in summer and those harbouring viral pathogens in fall. Five (5%) of the 104 study patients were admitted to the hospital from nursing homes, while all other patients were admitted from home.

Coexisting medical conditions were reported for 33 patients: Charlson Comorbidity Index score was 1 in 18, 2 in 2, 3 in 6, 4 in 2 and ≥ 5 in 6 cases. Three patients received antibiotic treatment (one patient with *Salmonella enterica *serovar (*S*.) Paratyphi, and two patients with blood cultures positive for *S. *Enteritidis). No fatalities were observed in patients of this study during their hospital stay.

Median length of hospital stay was 5.5 (1–34) days. In univariate analysis, length of stay was independent from the pathogen group and the patient's sex, but was significantly related to age and medical pre-conditions of patients. In multivariate analysis, the presence of any medical pre-condition remained the only significant predictor of a prolonged length of stay (OR 4,8; 95%CI 2,0–11,6) (table 1). As a continuous variable, Charlson Comorbidity score was also significantly related to a prolonged length of stay (OR 2,1 per index point; 95%CI 1,3–3,2).

**Table 1:** Predictors of a prolonged length of hospital stay in patients hospitalized with acute gastroenteritis (n = 96).

	Univariate analysis		Multivariate analysis	
		
	RR (95%CI)	p	OR (95%CI)	p
Age	1,6 (1,1–2,2)	0,008	1,9 (0,7–4,9)	0,2
Sex	1,2 (0,9–1,6)	0,2		
CCI ≥ 1^a^	2,0 (1,3–3,2)	<0,001	4,8 (2,0–11,6)	0,01
Bacteria^b^	1,0 (0,7–1,4)	1		
Viruses^c^	1,0 (0,7–1,4)	1		
Bacteria & viruses^d^	1,1 (0,8–1,7)	0,8		
Unknown aetiology	0,8 (0,5–1,3)	0,4		

^a ^CCI: Charlson comorbidity index.

^b ^*Campylobacter spp. *(28; including one co-infection with *B. hominis*), *Salmonella spp. *(16; including one co-infection with *B. hominis*), *Yersinia spp. *(6), *Shigella spp. *(2), enterohemorrhagic *E. coli *(1), *C. difficile *(3), atypical mycobacteria (2), *S*. Paratyphi (1).

^c ^Norovirus (16), rotavirus (5), and enterovirus (2).

^d ^*Salmonella spp. *and rotavirus (3) or enterovirus (2), *Campylobacter spp. *and rotavirus (2), enterovirus (1) or adenovirus (1), *C. difficile *and norovirus (1), atypical mycobacteria and norovirus (1), *Shigella spp. *and rotavirus (1), and *Yersinia spp. *and rotavirus.

### Non-infectious disease aetiology

In 8 (8%) patients, non-infectious aetiology of gastroenteritis was found: non-clostridium antibiotic-associated diarrhea (n = 4), alcohol-induced diarrhea (n = 2), one patient with diarrhea induced by non-steroidal anti-inflammatory drugs, and toxin-induced diarrhea in one case. Patients with non-infectious aetiology were excluded from further analysis.

### Microbiological findings

**Figure 1:**
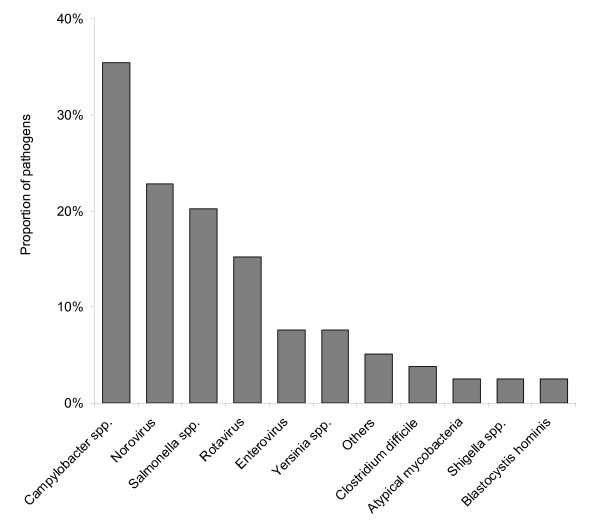
**Proportion (%) of pathogens as identified in 79 patients hospitalized with acute gastroenteritis.** Others: enterohemorrhagic *E. coli *(1), *S*. Paratyphi (1), adenovirus (1), *Giardia lamblia *(1).

In 79 (82%) of the remaining 96 patients whose stool samples were further investigated, at least one pathogen was found (figure 1). From the 26 pathogens tested 13 were detected (table 2). Among the patients with positive test results (n = 79), *Campylobacter spp. *was the pathogen most frequently diagnosed (28 patients, 35%) in our study cohort. Of these, 13 (46%) patients were exclusively diagnosed by serology, and 15 were diagnosed by positive stool culture. In patients diagnosed by serology, co-infections with viruses were detected in three patients (including rotavirus, norovirus, and adenovirus). In patients with positive stool culture for campylobacter (n = 15), *C. jejuni *was isolated in 11 patients, and *C. upsaliensis *was found in a single case. In three patients, no species differentiation was performed.

**Table 2:** List of pathogens included in study panel study, and frequency of detection.

not detected	detected in 10 patients	detected in > 10 patients
*Aeromonas spp.*		
*Plesiomonas spp.*		
Vibrio spp.	*Shigella ssp.*	
*Arcobacter butzleri*	Enterohemorrhagic *E. coli*	
*Listeria monocytogenes*	Adenovirus	*Salmonella spp.*
*Cyclospora cayetanensis*	*Blastocystis hominis*	*Campylobacter spp.*
*Isospora belli*	Mycobacteria	Norovirus
Cryptosporidia	*Giardia lamblia*	Rotavirus
*Entamoeba histolytica/dispar*	*Yersinia ssp.*	
*Balantidium coli*	Enterovirus	
Helminth eggs	*Clostridium difficile*	
Aichivirus		
Astrovirus		

Stool cultures from 16 (20%) patients yielded non-typhoidal *Salmonella spp.*; 12 of these isolates were serotyped as *S*. Enteritidis, three as *S*. Typhimurium, and one as *S*. Derby. In two patients harboring *S*. Enteritidis, blood-cultures were also positive.

Norovirus and rotavirus were detected by RT-PCR in 18 (23%) and 12 (15%) patients, respectively. Enterovirus was detected in six (8%) patients four of whom were co-infected by bacteria. Adenovirus was found in a single (1%) patient with a concomitant *Campylobacter spp. *infection.

*B. hominis *was found as a co-pathogen in two (3%) patients (*S. *Enteritidis and *Campylobacter spp.*). In three (4%) patients, tests for *C. difficile *toxins were positive; in two of these cases, a co-infection was detected (norovirus and *Salmonella *Typhimurium). A positive test result for *C. difficile *was significantly associated with the use of antimicrobials in the three months prior to hospital admission (RR 17; 95%CI 1.7–173; p = 0.03). All cases of yersiniosis (n = 6, 7%) were exclusively diagnosed by serology. In two patients, atypical mycobacteria (*M. avium-intracellulare*) were cultured; one patient was co-infected with *S. *Enteritidis and rotavirus, the other one with norovirus. The distribution of pathogens divided in etiological groups is shown in table 3. Higher age (by year) and lower frequency of stools were significantly associated with the detection of viral pathogens (p = 0.03).

**Table 3:** Characteristics of the patients, grouped by pathogens.

	n	Median Age (range)	% female	max. freq. stool/day
Bacteria^a^	42	40 (18–79)	48	10
Viruses^b^	23	65 (18–87)*	70	5*
Bacteria & viruses^c^	13	48 (20–79)	62	10
Unknown	17	39 (22–91)	65	8
Other^d^	1			

Total	96			

^a, b, c ^see table 1; ^d ^*G. lamblia*; * p > 0.05 in ANOVA (using mean values).

Patients admitted from nursing homes did not differ significantly from other patients with respect to clinical presentation, detected pathogens, and demographic variables.

### Mixed infections

In 17 of 79 cases (22%; 95% CI: 13–32%) more than one pathogen was found (two pathogens in 14 patients, three pathogens in three patients). Bacteria-viruses co-infections were detected in 13 (76%) of these patients, bacteria-parasites in two patients, bacteria-bacteria and viruses-viruses in single patients. Simultaneous infection was significantly more likely in patients with rotavirus infections (RR 3.6; 95% CI: 1.8–7.4; p = 0.004), enterovirus infections (RR 3.5; 95% CI: 1.7–7.4; p = 0.001), and salmonella infections (RR 2.5; 95%CI: 1.2–5.5; p = 0.02). The presence of co-infections caused by these pathogens was independent from age, sex, or presence of medical pre-conditions. Among patients diagnosed with rotavirus or *Salmonella spp. *infections, those with co-infections reported a significantly higher mean frequency of stools when compared to patients with single infections of the respective pathogen (12 vs. 4, p = 0.04; 12 vs. 6; p < 0.05). There was no significant association between mixed enterovirus infections and a higher stool frequency.

### Unknown disease aetiology

No etiologic agent or other cause for acute diarrhea was found in 17 (18%) patients. Of these, 11 (65%) patients were female, and the median age was 39 years. No seasonal pattern was found in cases with unknown aetiology. Failure to detect a pathogen was associated with use of antacids before admission (RR 2.7; 95%CI 1.1–6.4; p = 0.03).

## Discussion

Our study provides novel insights into the etiologies and characteristics of acute, community-acquired infectious gastroenteritis in adults presenting to an university hospital in an industrialized country. After excluding of non-infectious disease etiologies, a causative pathogen was identified in 82% of the patients. These results demonstrate that comprehensive microbiological analysis substantially reduces the diagnostic gap described in previous studies [4-7].

*Campylobacter spp. *and *Salmonella spp. *were the most common bacterial pathogens found in our cohort. This is consistent with earlier reports using a comparable study design [4,6,11,12]. In contrast to our results, however, the ratio of these two bacterial pathogens has previously been reported to be more or less balanced. The high proportion of *Campylobacter spp. *infections found in our study is most likely explained by the addition of serology for *Campylobacter *(i.e. seroconversion in CFT) as a diagnostic tool [13]. Although we used state-of-the-art culture methods, 46% of all patients with *Campylobacter spp. *infection were solely detected by serology (i.e., seroconversion). Similar findings have been reported from outbreaks with *Campylobacter spp.*, and the additional use of serology in investigations of outbreaks has been proposed [14,15]. Our results suggest that currently employed culture-based methods have a limited sensitivity also in hospital settings, and may significantly underestimate the incidence of *Campylobacter spp. *infection (and possibly prevent timely antibiotic treatment).

Viral pathogens were detected in approximately one-third of patients, with norovirus being the most common agent. This is a considerably higher proportion compared to those of previous studies conducted in other industrialized countries, where rotavirus and norovirus were found in less than 5% of patients [3,4,16], and may be explained by an increasing incidence of these emerging viral infections, the more frequent employment of sensitive detection methods (RT-PCR) in routine diagnostic of these pathogens in recent years, or both [17]. The increasing relevance of viral pathogens – in particular rotavirus – as a cause for severe gastroenteritis not only in children, but also in adults is of importance for both diagnostics and prevention of the disease in hospitalized adults, especially with respect to possible rotavirus vaccination campaigns.

In 22% of our patients more than one pathogen was detected. Although several studies emphasized the importance of concomitant infections in infantile gastrointestinal disease, prospective data on the relevance of co-infections in adults hospitalized with gastroenteritis are sparse, and dual infections have not been systematically investigated in this population so far. In addition, the interpretation of data on mixed viral-bacterial infections, is complicated by the fact that in many mixed infections one of the potential enteropathogens (e.g., *M. avium-intracellulare*) may not etiologically contribute to the gastroenteritis. However, in our study patients with mixed rotavirus or salmonella infections had a more severe course of disease (as indicated by a significantly higher frequency of stools than in patients with single pathogens), thus suggesting that mixed infections indeed contribute to the clinical presentation of gastrointestinal disease in adults.

Due to the limited sample size we were not able to look for other indicators of severe gastroenteritis in patients with mixed infections (e.g., admission to an intensive care unit, or death) that warrant further examination. Our findings, however, are of diagnostic and clinical relevance especially in patients with a severe course of viral gastroenteritis, since a second – potentially treatable – agent may enhance disease severity. In these cases, a positive result does not exclude other pathogens, and further diagnosis should be encouraged.

Unless there are epidemiological or clinical evidence for a specific pathogen, however, our results suggest that testing for community-acquired enteric pathogens in hospitalized adults in developed countries should initially include *Salmonella spp.*, *Campylobacter spp.*, Norovirus, and Rotavirus. The costs for this smaller pathogen panel were about € 180 in our study.

Though included in the laboratory protocols, we did not detect the "emerging" pathogens aichivirus or *Arcobacter butzleri *in any of the patients, suggesting that these pathogens were not important in our study population at the time of the investigation.

Almost one-third of patients in this study had coexisting medical conditions. This adds to the notion that patients with co-morbidities are at increased risk of developing a severe course of gastroenteritis that leads to hospitalisation [18]. Surprisingly, the duration of hospital stay was unrelated to the age of the patient or the aetiology of the infection, but was significantly longer in patients with a medical history. Future research and strategies to prevent severe gastroenteritis in adults for all groups of pathogens should primarily focus on patients with known co-morbidity.

Although our study provides one of the most thorough analyses of gastroenteritis in hospitalized adults thus far, it does have limitations. First, our study population may be different from other adult populations with gastroenteritis, e.g., because of regional variations in the incidence and prevalence of gastrointestinal pathogens. Our main findings, however, are unlikely to be affected by regional differences, since the sensitivity of stool cultures for *Campylobacter *and the increase of viral gastrointestinal infections that have been observed Europe-wide are not primarily related to geographic variations.

Second, we did not obtain paired serum samples for campylobacter serology from all patients, especially from patients with positive stool culture; future investigations should compare stool culture, serology, and other diagnostic methods (i.e. antigen tests, PCR) to allow the calculation of test-specific sensitivity and specificity.

## Conclusion

In conclusion, a pathogen was detected in more than four-fifths of adult patients hospitalized with acute gastroenteritis in this study. Culture-based methods for the diagnosis of *Campylobacter spp. *seem to miss a substantial proportion of infections, and should be flanked by serology, or replaced by other diagnostic tests. Clinicians should be aware of viral pathogens and mixed infections (i.e., viral-bacterial) as a cause of severe gastroenteritis in adults. Finally, in our study the duration of hospital stay was related to the presence of medical pre-conditions, but not to the etiological agent. This finding might help to develop clinical algorithms that support decision-making for in-patient vs. outpatient management.

## Competing interests

The authors declare that they have no competing interests.

## Authors' contributions

AJ, KS, and DW were involved in the study conception. AJ carried out the statistical analysis of the cohort study, and drafted the manuscript. RI, OL, and UBG were involved in the laboratory analyses for bacteria, and parasites. ES was in charge of the laboratory investigations for viral pathogens. JH, MZ, and TS were in charge of recruitment, examination, treatment and follow-up of the patients. All authors were involved in the interpretation and validation of the results. All authors read and approved the final manuscript.

## Pre-publication history

The pre-publication history for this paper can be accessed here:


